# Recognition of damage-associated molecular patterns related to nucleic acids during inflammation and vaccination

**DOI:** 10.3389/fcimb.2012.00168

**Published:** 2013-01-08

**Authors:** Nao Jounai, Kouji Kobiyama, Fumihiko Takeshita, Ken J. Ishii

**Affiliations:** ^1^Laboratory of Adjuvant Innovation, National Institute of Biomedical InnovationOsaka, Japan; ^2^Laboratory of Vaccine Science, WPI Immunology Frontier Research Center, Osaka UniversityOsaka, Japan

**Keywords:** PAMPs (pathogen-associated molecular patterns), DAMPs (damage-associated molecular patterns), nucleic acids, metabolites, innate immunity, DNA sensors, uric acid, vaccine adjuvant

## Abstract

All mammalian cells are equipped with large numbers of sensors for protection from various sorts of invaders, who, in turn, are equipped with molecules containing pathogen-associated molecular patterns (PAMPs). Once these sensors recognize non-self antigens containing PAMPs, various physiological responses including inflammation are induced to eliminate the pathogens. However, the host sometimes suffers from chronic infection or continuous injuries, resulting in production of self-molecules containing damage-associated molecular patterns (DAMPs). DAMPs are also responsible for the elimination of pathogens, but promiscuous recognition of DAMPs through sensors against PAMPs has been reported. Accumulation of DAMPs leads to massive inflammation and continuous production of DAMPs; that is, a vicious circle leading to the development of autoimmune disease. From a vaccinological point of view, the accurate recognition of both PAMPs and DAMPs is important for vaccine immunogenicity, because vaccine adjuvants are composed of several PAMPs and/or DAMPs, which are also associated with severe adverse events after vaccination. Here, we review as the roles of PAMPs and DAMPs upon infection with pathogens or inflammation, and the sensors responsible for recognizing them, as well as their relationship with the development of autoimmune disease or the immunogenicity of vaccines.

## Introduction

Host cells are equipped with numerous types of receptors to discriminate self from non-self. When cells are attacked by infectious pathogens, host cellular receptors such as Toll-like receptors (TLRs), nucleotide oligomerization domain (NOD)-like receptors (NLRs), retinoic acid-inducible gene-I (RIG-I)-like receptors (RLRs), C-type lectin receptors, and other non-classified receptors recognize pathogen-associated molecular patterns (PAMPs), small molecular motifs conserved amongst microbes. Through the recognition of PAMP molecules, innate immune responses are induced, and inflammatory cytokines are produced that aid in the elimination of the pathogens. However, in some circumstances host inflammatory responses can cause host cell death leading to tissue injury, and the release of host cellular components to the extracellular environment. These cellular components could be considered “messengers” for danger; they are also known as “damage-associated molecular patterns” (DAMPs). DAMPs include lipids, sugars, metabolites, and nucleic acids such as RNA and DNA species. DAMPs are important for the elimination of pathogens, but are also implicated in the development of autoimmune disease and chronic inflammatory disease, and are used as adjuvants for vaccines. Interestingly, high numbers of PAMP receptors also recognize endogenous DAMPs and can augment inflammatory responses against pathogens, whereas continuous inflammatory responses owing to impaired regulation of inflammatory signaling results in chronic inflammatory disease or autoimmune disease. Therefore, “bipolar sensors” for both PAMPs and DAMPs appear to be the mostly responsible for dysregulated inflammation. Here, we describe the various types of DAMPs and their receptors, with a special focus on nucleic acids as DAMPs.

## Lipid-related DAMPs

### Lipopolysaccharide (LPS)

A representative lipid for the induction of inflammatory responses is LPS, a PAMP present in gram-negative bacteria. Upon recognition by TLR4, LPS promotes the production of various inflammatory cytokines following bacterial infection (Table 1). However, Shi et al. reported that, TLR4 also recognizes endogenous fatty acids and can activate inflammatory responses in adipocytes and macrophages (Shi et al., 2006). In addition, TLR4-deficient mice developed reduced inflammatory cytokine production in response to a high fat diet (Shi et al., 2006). Previous studies have revealed that saturated fatty acids are released from hypertrophied adipocytes in the presence of macrophages, and that released fatty acids are sensed by macrophages in a TLR4-dependent manner, following excessive production of inflammatory cytokines such as tumor necrosis factor (TNF)-α (Suganami et al., 2007). Because the production of pro-inflammatory or inflammatory cytokines is dysregulated in obese adipose tissues, obesity can be thought of as a chronic inflammatory disease caused by fatty acids acting as DAMP molecules (Berg and Scherer, 2005).

**Table 1:** **Association of PAMP or DAMP sensors with autoimmune diseases**.

**Receptor**	**PAMP**	**DAMP**	**Autoimmune disease**
TLR1/TLR2	Lipopeptide	Serum amyloid A protein	Atherosclerosis, rheumatoid arthritis, Crohn's disease
TLR4	LPS	Fatty acid	Obesity
		Hyaluronic acid	Rheumatoid arthritis, sarcoidosis, systemic sclerosis, pancreatic cancer
NLRP3	Uric acid	Uric acid	Hyperuricemia, gout
		ATP	Unknown
RIG-I, MDA5, TLR7/8	Virus RNA	Immunocomplex of snRNPs	SLE
TLR9	Bacterial DNA	Self-DNA-containing immune complexes, histone	SLE
RAGE	−/?	HMGB1	SLE
DAI, IFI16, AIM2, H2B, RNA pol III	Bacterial DNA, Virus DNA	Self-DNA?	SLE?

### Serum amyloid a protein (SAA)

Some lipoproteins can also act as DAMP molecules. In 1982, Hoffman and Benditt revealed that the treatment of mice with LPS of *Salmonella typhosa* increased SAA levels (Hoffman and Benditt, 1982). According to several studies, SAA functions in cholesterol transport as well as in the production of proinflammatory cytokines, suggesting that SAA is a DAMP molecule that responds to bacterial endotoxins (Banka et al., 1995; He et al., 2003). In support of this, increased levels of SAA may be closely related to various diseases such as atherosclerosis, rheumatoid arthritis, and Crohn's disease (Chambers et al., 1983, 1987; Malle and De Beer, 1996). SAA binds to two receptors, TLR4 and TLR2, which also recognize bacterial PAMP molecules such as triacyl lipopeptides (in cooperation with TLR1), diacyl lipopeptides or lipoteichoic acids (together with TLR6) (Schwandner et al., 1999; Takeuchi et al., 2001, 2002; Cheng et al., 2008; Hiratsuka et al., 2008) (Table 1). Recently, Loser et al. showed direct evidence for the local production of the SAA molecules myeloid-related protein-8 (Mrp8) and Mrp14, which induced autoreactive CD8^+^ T cells and systemic autoimmunity through TLR4 signaling in mice (Loser et al., 2010). Taken together, these findings suggest that TLR4 may be a key receptor in the discrimination of lipid PAMPs from lipid DAMPs molecules, because promiscuous recognition of lipids via TLR4 unfortunately causes inflammatory disease. Although a consensus recognition structure for TLR4 has not yet been identified, antagonists of TLR4 signaling by lipid-DAMPs might be candidate drugs for the treatment of chronic inflammatory disease.

## Sugar-related DAMPs

Hyaluronic acid (HA) is a non-sulfated linear polysaccharide, and a major component of the extracellular matrix. Weigel et al. revealed that HA is induced and degraded during inflammatory responses and that it functions in immune cell activation or new blood vessel formation (Weigel et al., 1986). Interestingly, small molecular weight HA (sHA), produced by the degradation of HA during inflammation, can induce the maturation of dendritic cells (DCs) for pathogen elimination (Termeer et al., 2002). Bone marrow-derived DCs from mice expressing non-functional TLR4 could not be activated by sHA, while DCs from TLR2-deficient mice retained the ability for sHA-mediated activation. This suggests that sHA can act as a DAMP molecule signaling through TLR4 to induce DC maturation upon pathogen infection (Termeer et al., 2002). Consistent with this, excessive sHA levels appeared to be closely associated with inflammatory autoimmune diseases such as rheumatoid arthritis, sarcoidosis, systemic sclerosis, and pancreatic cancer (Hallgren et al., 1985; Witter et al., 1987; Sugahara et al., 2006; Yoshizaki et al., 2008) (Table 1).

## Metabolite-related DAMPs

### Uric acid

Uric acid is a metabolite of purine nucleotides and free bases in humans and other primates, and it functions as an antioxidant to protect erythrocyte membranes from lipid oxidation (Kellogg and Fridovich, 1977). However, it was previously shown that soluble uric acid-induced inflammatory cytokines such as monocyte chemoattractant protein-1 in rat vascular smooth muscle cells (Kanellis et al., 2003). Shi et al. also reported that uric acid is produced in ultraviolet-irradiated BALB/c 3T3 cells, and activates DCs (Shi et al., 2003). In addition, high levels of uric acid in the blood are associated with the development of hyperuricemia and gout (Johnson et al., 2005), suggesting that it acts as a DAMP during cell injury and can induce inflammatory responses that are related to autoinflammatory diseases such as gout (Table 1).

Receptors that recognize uric acid have been reported and Liu-Bryan et al. revealed that TLR2, TLR4, and their adaptor molecule MyD88 are important for uric acid-mediated inflammation (Liu-Bryan et al., 2005). In contrast, the uric acid-mediated activation of DCs was shown to be TLR4-independent, suggesting the possible existence of other receptors that recognize uric acid in addition to TLR2 and TLR4 (Shi et al., 2003). To solve this question, Martinon et al. demonstrated that uric acid could be sensed by another receptor, NOD-like receptor family, pyrin domain-containing 3 (NLRP3), and induced to produce interleukin (IL)-1β through caspase-1 activation (Martinon et al., 2006). NLRP3 is a member of the NLR family, and a component of the inflammasome, a platform that induces IL-1β and IL-18 production. NLRP3 senses various types of pathogen infections or irritants such as *Candida albicans*, *Legionella pneumophila*, *Listeria monocytogenes*, *Malaria hemozoin*, alum, silica, and asbestos as well as uric acid (Kanneganti et al., 2006; Martinon et al., 2006; Dostert et al., 2008, 2009; Eisenbarth et al., 2008; Gross et al., 2009). Collectively, these results revealed that NLRP3 is a promiscuous receptor that senses PAMPs and DAMPs and can induce inflammatory responses.

### Adenosine triphosphate (ATP)

ATP is an essential purine base required for almost all physical responses such as glucose metabolism, muscle contraction, biosynthesis, and molecular transfer. However, extracellular ATP from injured cells or non-apoptotic cells also serves as a danger signal through the activation of NLRP3 and caspase-1 (Communi et al., 2000). Previous detailed research has shown the importance of other ion channel molecules, namely, P2X7 and pannexin-1, in inducing extracellular ATP-mediated caspase-1 activation following IL-1β maturation (Ferrari et al., 2006; Kanneganti et al., 2007). The formation of the NLRP3 inflammasome requires an adaptor molecule, apoptosis-associated speck-like protein containing a carboxy-terminal caspase recruitment domain (ASC). ASC-deficient mice cannot activate caspase-1 and thus do not produce mature IL-1β following exposure to large amounts of ATP, suggesting that ATP-mediated IL-1β production is dependent on the NLRP3 inflammasome (Mariathasan et al., 2004). However, although extracellular ATP has been suggested to act as a DAMP molecule, there is no correlation between high amounts of extracellular ATP acting as DAMPs *in vitro* and physiological conditions *in vivo*. Eckle et al. suggested that most extracellular ATP might be immediately hydrolyzed by ectonucleotidases (Eckle et al., 2007). Taken together, investigation into the roles of extracellular ATP in inducing pathological and immune responses *in vivo* may provide important clues regarding the mechanism underlying inflammation induction by DAMP molecule recognition or in the development of inflammatory diseases.

## Nucleic acid-related DAMPs

### Unmethylated CpG motif and genomic DNA

As described above, uric acid and ATP are products of purine metabolism. Nucleic acids such as adenine or guanine are also purine metabolites. Nucleic acids exist in all organisms including pathogens, and function as a store of genetic information for protein translation and synthesis. Bacterial genomic DNA can be recognized as a PAMP, as it contains unmethylated CpG motifs whose frequency is higher in genomic DNA derived from pathogens compared with that of vertebrates. The earliest research related to bacterial genomic DNA as PAMPs was reported more than hundred years ago. Bruns et al. investigated heat-killed gram-negative or gram-positive bacteria as an immunotherapeutic agent termed Coley's toxin, for cancer (Swain, 1895). Although LPS is a major factor in mediating anti-tumor effects, other factors may be connected with its physiological function, as gram-positive bacteria do not express LPS. A hundred years on from the discovery of Coley's toxin, several studies have shown that bacterial DNA can activate natural killer (NK) cells or B cells, suggesting that the bacterial genomic DNA in Coley's toxin could contribute to its anti-tumor activity by stimulating NK cells (Shimada et al., 1986; Messina et al., 1991). Krieg et al. further revealed that bacterial genomic DNA contains unmethylated CpG motifs that can stimulate B cells and NK cells, and induce inflammatory cytokine production. Interestingly, methylated bacterial DNA failed to stimulate immune cells, indicating that unmethylated CpG motifs may act as PAMP molecules (Krieg et al., 1995; Klinman et al., 1996). However, whether genomic DNA containing methylated CpG motifs is incapable of innate immune activation remains controversial. In 1962, Glasgow et al. reported that ultraviolet-inactivated vaccinia virus, a DNA virus, resulted in IFN production in mouse cells (Glasgow and Habel, 1962). In addition, Suzuki et al. showed that viral DNA, vertebrate DNA and bacterial DNA induced the upregulation of major histocompatibility complex (MHC) class I expression and the type I IFN-related activation of transcription factors such as STAT3 in rat thyroid cells, suggesting that genomic DNA also activates innate immune signaling in a CpG-motif-independent manner (Suzuki et al., 1999). Interestingly, the structure of DNA strongly affects DNA-mediated innate immune activation. Double-stranded, right-handed B-form DNA, but not the left-handed Z-form DNA, strongly induced type I IFN production. Genomic DNA has a high content of B-form DNA, indicating that it may also function as a PAMP or DAMP (Ishii et al., 2006). Mitochondrial DNA has been also reported to function as a DAMP molecule. Zhang et al. reported that cellular injury caused the release of mitochondrial DNA, and induced systemic inflammatory responses via p38 MAPK activation in a TLR9-dependent manner. In addition, trauma patients had higher amounts of mitochondrial DNA than did healthy volunteers, suggesting that mitochondrial DNA could be considered a marker of inflammatory disease (Zhang et al., 2010). When the clearance of mitochondrial DNA by autophagy was inhibited, IL-1β production was augmented via the NLRP3 inflammasome to activate caspase-1, indicating that the amount of mitochondrial DNA DAMP activity is regulated by autophagy to suppress erroneous activation of innate immunity (Nakahira et al., 2011). Indeed, it was revealed that autophagy negatively regulates RNA-mediated type I IFN production, possibly to maintain cellular homeostasis (Jounai et al., 2007).

### Correlation between autoimmune disease and DNA DAMPs

Both DNA and RNA can function as PAMPs and DAMPs, and are closely connected with inflammatory responses and the development of inflammatory disease. Direct evidence for DNA acting as a DAMP was shown using DNase-deficient mice. DNase I is present in extracellular compartments such as the sera and urine, and functions to degrade single-stranded DNA (ssDNA), double-stranded DNA (dsDNA), or chromatin, which are released from damaged or necrotic cells. Napirei et al. constructed DNase I-deficient mice, and reported that they presented with the classical symptoms of systemic lupus erythematosus (SLE) and glomerulonephritis (Napirei et al., 2000). In addition, DNase II deficient mice showed a similar phenotype to DNase I knockout mice. DNase II in the lysosomes of macrophages degrades DNA from apoptotic cells or nuclear genome DNA from liver erythroblasts. Interestingly, DNase II-deficient mice presented with lethal anemia owing to high levels of type I IFN production, caused by the accumulation of non-degraded genomic DNA in liver macrophages (Yoshida et al., 2005). In support of this, *DNase II* and *IFNRa/b* double knockout mice showed a non-lethal phenotype, but developed rheumatoid arthritis-like symptoms (Kawane et al., 2006), which could be attenuated by anti-TNF-α antibody treatment. This suggested that the accumulation of genomic DNA in macrophages induced inflammatory cytokines, including type I IFNs and TNF-α, and the synergistic action of these inflammatory cytokines resulted in lethal systemic inflammation (Kawane et al., 2006). Furthermore, studies on DNase III, also known as TREX1, also revealed that DNA could function as a DAMP. TREX1 is the major 3′ → 5′ DNA exonuclease for DNA editing in DNA replication or DNA repair. Morita et al. showed that *trex1*-deficient mice had a reduced survival rate owing to high susceptibility to inflammatory myocarditis, although null mice showed no spontaneous mutations or tumor development (Morita et al., 2004). To explain why *trex1*-deficient mice develop inflammatory myocarditis, Crow et al. demonstrated that the mutation in the *trex1* gene that abolished TREX1 enzyme activity was responsible for the development of Aicardi-Goutieres syndrome (AGS), a severe neurological brain disease with high levels of IFN-α in cerebrospinal fluid or serum, suggesting that TREX1 is a suppressor of DNA DAMP-mediated inflammatory responses (Crow et al., 2006). Furthermore, it was previously shown that the abolishment of interferon regulatory factor 3 (IRF3) or IFN-α receptor 1 ameliorated the AGS symptoms in *trex1*-deficient mice (Stetson et al., 2008). Collectively, these findings suggest that the dysregulation of self-DNA results in severe inflammatory responses such as high levels of type I IFNs leading to autoinflammatory disease.

## Nucleic acid sensors

**Figure 1:**
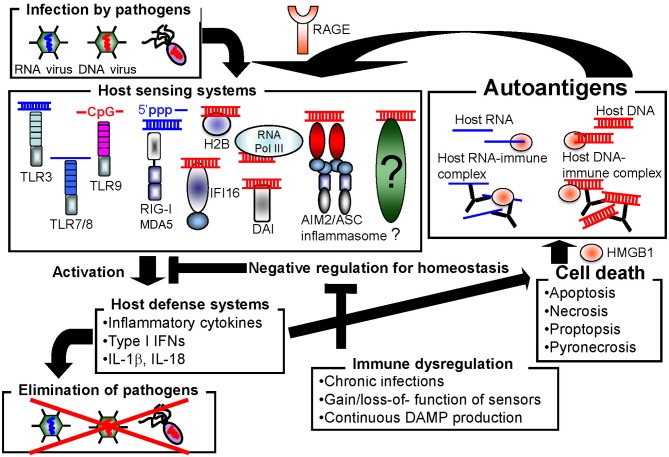
**Autoimmune disorders may be induced by promiscuous sensing of nucleic acids**.

Host cells are equipped with numerous types of receptors to recognize nucleic acids as PAMPs or DAMPs. These receptors function to protect the host from pathogen infection, but may also cause autoimmune disorders by inducing the constitutive activation of inflammatory responses (Figure 1). In this section, we introduce the well-characterized nucleic acid sensors.

### TLRs

**Figure 2:**
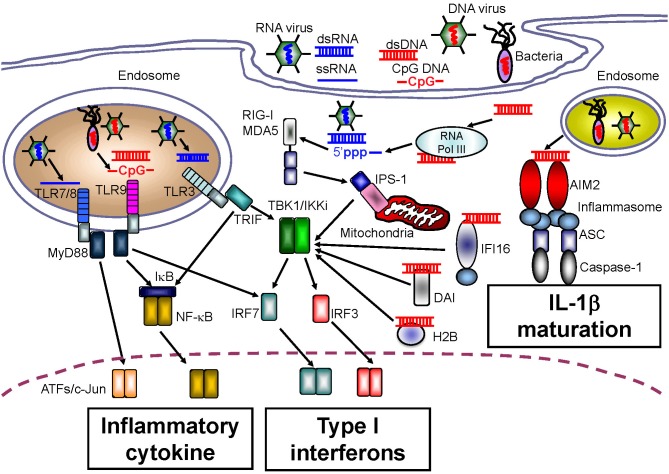
**Intracellular sensors for nucleic acids**.

A large body of research exists demonstrating the TLR-mediated sensing of nucleic acids. TLR3 preferentially senses double-stranded RNA (dsRNA) species, which can originate from some viruses, and TLR3 is associated with induction of innate immunity in response to infection with West Nile virus, respiratory syncytial virus, and encephalomyocarditis virus (Wang et al., 2004; Groskreutz et al., 2006; Hardarson et al., 2007) (Figure 2). In addition, artificial dsRNA, poly (I:C), has been well-characterized as a ligand for TLR3. Although pathogen-related dsRNAs act as PAMPs, Kariko et al. reported that host messenger RNA could be sensed by TLR3 to induce inflammatory responses (Kariko et al., 2004). RNA released from necrotic cells can also elicit type I IFN production, suggesting that host RNA might function as a DAMP upon cellular injury (Kariko et al., 2004).

TLR7 and TLR8 recognize single-stranded RNA (ssRNA), and induce anti-viral innate immune responses against influenza virus or vesicular stomatitis virus (Lund et al., 2004) (Figure 2). Regardless of their common ligands, the cellular and tissue distribution of TLR7 expression is in contrast to that of TLR8. Human TLR7 is highly expressed in plasmacytoid DCs that preferentially induce type I IFN production, and is expressed at lower levels in myeloid cells. Conversely, the level of TLR8 expression is higher in monocytes and in monocyte-derived DCs than in plasmacytoid DCs (Hornung et al., 2002). Furthermore, mouse TLR8 did not respond to ssRNA, but human TLR8 did, suggesting that TLR8 might be inactivated in mice, although several papers have also linked mouse TLR8 with neuronal apoptosis and autoimmunity (Heil et al., 2004; Gorden et al., 2006; Ma et al., 2006).

In addition to the recognition of PAMPs, Vollmer et al. revealed that promiscuous recognition through TLR7 or TLR8 causes the development of SLE with high levels of type I IFNs and TNF-α production (Vollmer et al., 2005). Because the sera from SLE patients contains high levels of autoantibodies against self-antigens, such as small nuclear ribonucleoprotein particles (snRNPs) including ssRNA, TLR7, or TLR8 could recognize the immunocomplex of snRNPs with autoantibodies thorough Fc receptor-mediated internalization (Vollmer et al., 2005). Interestingly, TLR7 appears to be a specific sensor for the induction of type I IFN production from plasmacytoid DCs, whereas TLR8 is specific for TNF-α production from monocytes in SLE patients, suggesting that plasmacytoid DCs and monocytes collaborate to develop inflammatory responses in SLE via distinct sensors.

TLR9 senses ssDNA containing unmethylated CpG motifs. Previous studies have revealed that TLR9 recognizes genomic DNA from pathogens such as murine cytomegalovirus and Herpes simplex virus type 1 or type 2 as PAMPs (Hemmi et al., 2000; Lund et al., 2003; Krug et al., 2004a,b) (Figure 2). With regard to the development of autoinflammatory disease, TLR9 has been also been reported to recognize self-antigens complexed with autoantibodies. Leadbetter et al. revealed that autoreactive B cells were activated by a chromatin-autoantibody complex in a TLR9- and MyD88-dependent manner (Leadbetter et al., 2002). In addition, self-DNA-containing immune complexes, which are a well-characterized marker for SLE, were recognized by TLR9 through FcγRIIA-mediated internalization in plasmacytoid DCs (Means et al., 2005). Thus, immune complexes containing self-DNA may signal as DAMPs through TLR9, although extracellular receptors such as FcγRIIA may be required for the delivery of autoimmune complexes to the TLR9-localizing compartment.

As described previously, the subcellular localization of TLRs is important for the recognition of DNA, because TLR3, 7, 8 and 9 localize to the endosomal compartment. Previous studies identified three adaptor molecules, Unc93B1, PRAT4A, and gp96, which are important for the trafficking of TLRs to sites for sensing their ligands. Unc93B1 functions to control the trafficking of TLRs 3, 7, and 9 from the endoplasmic reticulum (ER) to the endosome. PRAT4A is localized in the ER and acts as a regulator of the subcellular distribution of most TLRs except for TLR3. Gp96 is a member of the heat shock protein (HSP) 90 family, and resides in the ER where it controls the maturation of TLRs 2, 4, 5, 7, and 9 (Saitoh and Miyake, 2009). Because TLR7 and TLR9 are regulated by the same molecular machinery, the crosstalk between TLR7 and TLR9 may affect the sensing of auto-nucleic acids and the development of autoinflammatory disease. Christensen et al. showed that a deficiency of TLR9 results in malignant symptoms in a mouse model of lupus, despite the levels of antibody production specific for DNA and chromatin being down-regulated (Christensen et al., 2005). In contrast, TLR7-deficient mice developed attenuated lupus symptoms (Christensen et al., 2006). In addition, a recent study revealed that TLR9 suppressed the progression of autoinflammatory disease by antagonizing TLR7, suggesting that TLR9 counteracts TLR7 upon the recognition of self-immunocomplexes containing ssRNA or ssDNA (Nickerson et al., 2010). To support the interaction between TLR7 and TLR9 upon the development of autoimmune disease, Fukui et al. generated Unc93B1^D34A/D34A^ knock-in mice to show that TLR9 competes with TLR7 for binding to Unc93B1 in the healthy state, while TLR7 is constitutively activated upon autoinflammatory responses because TLR9 has a lower affinity for the Unc93B1-like Unc93B1^D34A/D34A^ mutant (Fukui et al., 2011).

### RIG-I-like receptors (RLRs)

Although TLRs can sense both non-self and self nucleic acids, fibroblasts, and endothelial cells that do not express TLRs also produce type I IFNs in response to infection with pathogens, indicating the existence of other receptors that sense nucleic acids. Yoneyama et al. determined that a cytoplasmic DExD/H box RNA helicase, RIG-I, senses infection by RNA viruses as well as artificial dsRNA, and induces innate antiviral immune responses mediated by type I IFNs (Yoneyama et al., 2004) (Figure 2). In addition to RIG-I, melanoma differentiation factor-5 (MDA5) and laboratory of genetics and physiology-2 (LGP2) were also identified; these receptors were classified as RLRs because their protein structures were similar to that of RIG-I (Yoneyama et al., 2005). To induce an anti-pathogen immune response, a CARD domain in RIG-I and MDA5 transmits down-stream signals through homophilic interactions with the CARD adaptor molecule, IFN-β promoter stimulator-1 (IPS-1, also known as MAVS, Cardif, or VISA) (Kawai et al., 2005; Meylan et al., 2005; Seth et al., 2005; Xu et al., 2005). The function of LGP2 is controversial. Some *in vitro* studies showed that LGP2 negatively regulates RIG-I- or MDA5-mediated innate immune responses by competing for binding with their RNA ligands (Yoneyama et al., 2005; Bamming and Horvath, 2009). However, *in vivo* studies using *lgp2*-deficient mice revealed that LGP2 is a cofactor of RLR-mediated innate immune signaling (Venkataraman et al., 2007; Satoh et al., 2010).

RLRs sense pathogen-derived RNA species as PAMPs to induce type I IFN production, while MDA5 has been detected as an autoantigen in clinically amyopathic dermatomyositis patients (Sato et al., 2009; Nakashima et al., 2010). Although it is not clear how extracellular MDA5 is produced, the accumulation of immunocomplexes containing MDA5 is a marker for the frequency of rapidly progressive interstitial lung disease (Sato et al., 2009; Nakashima et al., 2010). Accompanying these observations, loss of function single nucleotide polymorphisms have been found in RIG-I and IPS-1 that are closely related to the development of autoimmune disease (Pothlichet et al., 2011), suggesting that inhibition of RLR signaling may be important in the progression of autoimmune disease. However, as described earlier, excessive production of inflammatory cytokines including type I IFNs appears to result in autoinflammatory disease. In contrast, the dysfunction of RLRs induces poor type I IFN production, but leads to autoimmune disease (Nakashima et al., 2010; Pothlichet et al., 2011). One possibility to explain this phenomenon is that non-functional RLRs result in an increased susceptibility against various types of virus infections, and the subsequent virus-mediated cell death may cause the release of DAMPs and signaling through DAMP receptors. Support this possibility, the loss of MDA5 function increased the susceptibility of beta cells to viral infection with picornavirus or encephalomyocarditis virus-D, and resulted in type 1 diabetes, whose types of diabetes are often caused by virus infection or autoimmunity (Colli et al., 2010; McCartney et al., 2011). Further analyses are required to elucidate the cross-talk between RLR signaling and the development of autoimmune disease.

### Absent in melanoma 2 (AIM2)-like receptors (ALRs)

Although various NLR family members that can induce the activation of caspase-1 and maturation of IL-1β, IL-18, and IL-33 in response to a wide range of PAMP and DAMP molecules have been identified, no sensor of intracellular dsDNA for IL-1β maturation has been identified. However, four research groups concurrently reported a role for the novel intracellular DNA sensor, AIM2, in the activation of caspase-1 following IL-1β production (Burckstummer et al., 2009; Fernandes-Alnemri et al., 2009; Hornung et al., 2009; Roberts et al., 2009). AIM2 belongs to a family of hematopoietic interferon-inducible nuclear proteins with a 200-amino acid repeat (HIN-200), known as the p200 or PYHIN family. Currently, four HIN-200 family molecules have been identified in humans, and six in mice. HIN-200 family molecules share similar structural features, including a pyrin domain at the NH_2_ terminus, and a HIN-200 domain at the COOH terminus. Similar to the role of NLRP3 in IL-1β production, AIM2 causes oligomerization of the inflammasome upon DNA binding. The AIM2 inflammasome recruits ASC, an essential adaptor molecule, and induces NLRP3 inflammasome formation through homophilic interactions between the pyrin domain in AIM2 and that in ASC (Figure 2). The importance of the AIM2 inflammasome upon PAMP recognition has been confirmed by infection experiments using *aim2*-deficient macrophages infected with *Francisella tularensis*, *L. monocytogenes*, vaccinia virus, herpes simplex virus-1 and mouse cytomegalovirus (Fernandes-Alnemri et al., 2010; Rathinam et al., 2010).

A second ALR, interferon-inducible protein 16 (IFI16) in humans (a homologue of p204 in mice), has been also investigated as an intracellular dsDNA sensor. However, while AIM2 induces IL-1β production in response to intracellular dsDNA binding, IFI16 is a sensor for type I IFN production upon recognition of intracellular dsDNA (Unterholzner et al., 2010). Although IFI16 also contains a pyrin domain, the pyrin in IFI16 is quite distinct from that in AIM2 as it has a lower affinity for ASC. Consistent with these different features of pyrin, IFI16-mediated type I IFN production upon intracellular dsDNA stimulation was not affected by ASC deficiency, suggesting that the two HIN-200 family molecules regulate both IL-1β and type I IFN production upon the recognition of intracellular dsDNA (Unterholzner et al., 2010). Although AIM2-mediated signaling appears to be distinct from IFI16-mediated type I IFN production, recent research has revealed that IFI16 negatively regulates the AIM2-mediated activation of caspase-1 (Veeranki et al., 2011). As increased inflammatory cytokine production is closely related to the development of autoinflammatory disease, the regulation between AIM2-mediated innate immune signaling and IFI16 might be deregulated in patients with autoimmune disease.

Roberts et al. identified p202 and AIM2 as cytosolic DNA binding proteins in mice. p202 is another ALR molecule without a pyrin domain, indicating an inability to bind ASC for inflammasome formation (Roberts et al., 2009). p202 appears to be a negative regulator for AIM2-mediated signaling, as the reduction of p202 results in higher AIM2-mediated activation of caspase-1 in response to intracellular DNA. However, elevated levels of p202 have been reported to induce SLE-like symptoms in mice (Rozzo et al., 2001). Interestingly, p202 levels are varied among mouse species, while AIM2 is expressed at the same level, indicating that p202 expression is tightly correlated to SLE development. Furthermore, Ravichandran et al. revealed that ablation of the *aim2* gene leads to higher expression of p202 and type I IFNs in mice, and *aim2*-deficient mice are prone to SLE (Panchanathan et al., 2010). Taken together, these findings suggest that mouse p202 might be homologous to human IFI16. In support of this, expression levels of IFI16 and anti-IFI16 autoantibodies were dramatically increased in SLE patients, indicating that IFI16 has similar features to p202 (Mondini et al., 2006).

A recent article described a correlation between psoriasis symptoms and AIM2 activation. Psoriasis is a chronic autoinflammatory disease caused by increased IL-1β production leading to Th17 cell maturation (Ghoreschi et al., 2010). Dombrowski et al. observed increased levels of cytosolic DNA fragments in skin lesions from psoriatic patients, which could be sensed by AIM2 (Dombrowski et al., 2011). Interestingly, those DNA fragments, which might be released from skin lesions in psoriatic patients, were internalized through binding to the antimicrobial peptide LL-37 (Dombrowski et al., 2011). Previous studies have shown that the complex of self-DNA with LL-37 can activate plasmacytoid DCs to produce type I IFNs, and complex-mediated type I IFN production is closely related with skin lesion development in psoriasis (Nestle et al., 2005; Lande et al., 2007). AIM2 is an interferon-inducible gene, suggesting that LL-37 complexes with self-DNA activate plasmacytoid DCs to produce type I IFNs, and that the subsequent upregulation of AIM2 leads to IL-1β production, and finally, psoriatic skin lesions occur because of the increased levels of type I IFN production as well as IL-1β production.

### High mobility group box 1 (HMGB1)

HMGB1 has been reported to be a major DAMP molecule. Goodwin et al. first identified HMGB1 from calf thymus chromatin as a non-histone DNA-binding protein (Goodwin et al., 1973). However, Wang et al. showed that a mouse macrophage cell line released HMGB1 in response to LPS stimulation. In addition, LPS-treated mice developed increased serum levels of HMGB1, similar to human patients with sepsis, suggesting that HMGB1 is a DAMP molecule in regard to sepsis symptoms (Wang et al., 1999). Accumulating evidence suggests that cellular injury results in the release of HMGB1 leading to inflammation (Abraham et al., 2000; Scaffidi et al., 2002). Consistent with these observations, numerous studies have showed a correlation between HMGB1 and autoimmune/inflammatory diseases such as atherosclerosis, diabetes, SLE, rheumatoid arthritis and Sjögren syndrome (Taniguchi et al., 2003; Porto et al., 2006; Urbonaviciute et al., 2008; Devaraj et al., 2009).

As described previously, higher serum levels of immunocomplexes of self-DNA with autoantibodies is a hallmark of SLE. Previous research has shown that HMGB1 is also contained in immunocomplexes and can elicit inflammatory cytokine production, suggesting that HMGB1 may be a carrier of DNA DAMPs (Tian et al., 2007; Urbonaviciute et al., 2008). Furthermore, HMGB1 appears to promiscuously bind numerous molecules such as LPS, IFN-γ, IL-1β, and CXCL12 to induce synergistic physiological responses (Sha et al., 2008; Youn et al., 2008; Campana et al., 2009). Moreover, HMGB1 can sense pathogen-derived nucleic acids, which induce type I IFN production (Yanai et al., 2009). Collectively, HMGB1 might be a promiscuous carrier that enhances innate immune responses against PAMPs and DAMPs.

The receptors for HMGB1 have been investigated, but are still controversial. A well-studied receptor for HMGB1 is the receptor for advanced glycation end products (RAGE). Similar to HMGB1, RAGE is a promiscuous receptor that can bind to various ligands including DNA, RNA, SAA protein, HSPs and prion protein, suggesting that RAGE may sense a variety of DAMP molecules in an HMGB1-dependent or -independent manner (Sims et al., 2010). Experiments with *rage*-deficient mice revealed that HMGB1-mediated DNA sensing requires RAGE for internalization of DNA complexes to produce type I IFNs via TLR9 (Tian et al., 2007). Interestingly, RAGE could associate with TLR9 upon recognition of the A type of CpG-HMGB1 complex, indicating a possible function for RAGE as a bridge molecule between the extracellular HMGB1-DNA complex and the TLR9 compartment (Tian et al., 2007). In contrast to this observation, nucleosomes could sense HMGB1 complexes independently of RAGE. Instead of RAGE, TLR2 appears to be important for the recognition of HMGB1-nucleosome complexes, suggesting that the sensing machinery of the HMGB1-nucleosome complex might be distinct from that of the HMGB1-DNA complex, as the HMGB1-nucleosome complex could not elicit production of type I IFNs even though TNF-α or IL-10 were induced (Urbonaviciute et al., 2008). Furthermore, recent research identified a novel ligand for RAGE, complement C3a, that binds human stimulatory CpG DNA to induce type I IFNs in an HMGB1-independent manner. This suggests that RAGE-mediated DNA sensing may involve numerous ligands (Ruan et al., 2010). Although there are many varieties of HMGB1- or RAGE-mediated DNA recognition, both molecules are strongly associated with the induction of inflammation and the development of chronic inflammatory disease.

### DNA-dependent activator of IFN-regulatory factors (DAI)

DAI has been identified as a molecule that recognizes intracellular DNA. Previous studies have revealed that DAI senses Z-type DNA; however, it may also bind to B-type DNA and induce type I IFN production through associations with TBK1 and IRF3 (Takaoka et al., 2007). Interestingly, DAI-deficient mice responded normally to cytosolic dsDNA stimulation, suggesting that DAI may function as one of a number of DNA sensors in a cell type-specific fashion (Ishii et al., 2008). Currently, the function of DAI is controversial, although the genetic adjuvanticity of DAI has been shown to induce strong cytotoxic T cell responses (Lladser et al., 2011). Although the ability of DAI to recognize DNA DAMPs has not been determined yet, DAI might be a link between the development of autoimmune disease and host DNA immune complexes.

### Histones

Histone H2B (H2B) is a component of chromatin, and Kobiyama et al. identified that H2B also functions to sense intracellular dsDNA. Previous reports showed that histones act as DAMPs, and that excessive intracellular dsDNA induces type I IFNs through H2B (Kobiyama et al., 2010). In confirmation of this, H1 or H2 are released from the nucleus after DNA damage, and are translocated to mitochondria following the induction of apoptosis. In addition, H1, H2A, and H2B may act as antimicrobial proteins in certain animals, suggesting that H2B is an intracellular dsDNA sensor that recognizes dsDNA PAMPs and DAMPs (Kawashima et al., 2011). Histones may be related to autoimmune diseases as anti-histone antibodies were detected in patients with such diseases. Further analyses are required to clarify the relationship between histones and autoimmune disease.

### Ku70

Ku70 functions in DNA repair, V(D)J recombination and in retaining the telomere. Zhang et al. showed that various DNA species-induced the production of type III interferon, IFN-λ1, and identified Ku70 as a novel DNA sensor by pull-down assay from the nucleus compartment (Zhang et al., 2011a). While other DNA sensors are important for the production of type I IFNs, Ku70 appears to be important for type III IFN production through IRF1 and IRF7. Furthermore, Ku70-mediated type III IFN production is restricted when the length of intracellular DNA stimuli is greater than 500 base pairs.

### RNA polymerase III

As described above, RIG-I senses intracellular RNA species, but may also recognize intracellular dsDNA. siRNA treatment of a human hepatoma cell line, Huh7, suppressed dsDNA-mediated type I IFN production. Subsequently, Chiu et al. showed that RIG-I senses the transcribed RNA byproducts of DNA templates that are generated by RNA polymerase III (as is the case for poly(dA·dT)·poly(dT·dA) and EBV genomic DNA) and induces production of type I IFNs (Chiu et al., 2009). An inhibitor of RNA polymerase III suppressed DNA-mediated type I IFN production, suggesting that RNA polymerase III is a distinct DNA sensor. However, RNA polymerase III-mediated dsDNA sensing is restricted to sequences of DNA stimuli containing less dA·dT than dG·dC.

### DHX9 and DHX36

Although the DExD/H box RNA helicase family contains RIG-I and MDA5, which function as RNA sensors, recent reports have revealed a similar RNA helicase family of molecules (DExDc family) that contain DHX9 and DHX36, which function as ssDNA sensors in plasmacytoid DCs (Kim et al., 2010). Interestingly, while DHX36 senses CpG-A, DHX9 senses CpG-B in a MyD88-dependent manner. This may suggest that ssDNA PAMPs or DAMPs are recognized by either DHX9 or DHX36, but recent research has shown that DHX9 collaborates with IPS-1 to recognize dsRNA in myeloid DCs, indicating the promiscuous sensing of DHX9 (Zhang et al., 2011b).

### Leucine-rich repeat flightless-interacting protein 1 (Lrrfip1)

Some sensor molecules such as TLRs or NLRs share common molecular patterns, such as leucine rich repeats (LRRs), which are important for ligand recognition or protein–protein interactions. An LRR-containing molecule, Lrrfip1, has been reported to sense intracellular DNA or RNA (Yang et al., 2010). Interestingly, whereas other DNA sensors often regulate type I IFN-related transcription factors such as IRF3/7 or caspase-1 to induce maturation of IL-1β, Lrrfip1 stimulates β-catenin and CBP/p300 to enhance *ifnb1* transcription, indicating a novel pathway involving β-catenin for type I IFN production upon cytosolic DNA sensing. Because Wnt/β-catenin signaling is also linked to tumor development, further analyses may identify the machinery involved in the regulation of type I IFN signaling by Lrrfip1 under tumor development.

### STING (stimulator of interferon genes protein)

The major function of MHC class II is antigen presentation, while monoclonal antibodies against MHC class II can cause cell activation or apoptotic cell death. Jin et al. identified a novel tetraspanin family molecule, MPYS, associated with MHC-II-mediated cell death (Jin et al., 2008). Three research groups performing cDNA library screening to identify molecules associated with activation of the type I IFN promoter identified the same molecule, STING (also known as MITA, and ERIS). STING is a novel adaptor molecule that activates innate immune signaling mediated by intracellular nucleic acid stimuli (Ishikawa and Barber, 2008; Zhong et al., 2008; Sun et al., 2009). Surprisingly, the Barber research group further revealed that STING is essential for the induction of type I IFN production following sensing of cytosolic dsDNA, using STING-deficient mice. Based on their imaging analysis, STING appears to localize to the ER during the steady state, but translocates to the Golgi apparatus upon intracellular dsDNA stimulation to activate down-stream molecules such as TBK1. This suggests that STING is an essential adaptor molecule for cytosolic dsDNA-mediated type I IFN production in mice.

Cyclic-di-GMP and c-di-AMP are small molecules that function as second messengers and are important for cell survival, differentiation, colonization, and biofilm formation. Recent research has revealed that the cytosolic delivery of c-di-GMP or c-di-AMP-induced type I interferon (IFN) production from bone marrow macrophages, suggesting that c-di-GMP and c-di-AMP are bacterial PAMP molecules (McWhirter et al., 2009; Woodward et al., 2010). As type I IFN production by c-di-GMP or c-di-AMP requires their internalization, live invasive bacteria possibly produce these second messenger molecules after internalization into cells.

Recent reports have revealed that STING is a direct sensor of bacterial second messenger molecules, such as c-di-GMP or c-di-AMP (Burdette et al., 2011; Jin et al., 2011). This indicates the novel possibility that cytosolic dsDNA stimulation might produce c-di-GMP/c-di-AMP or related molecules that can be sensed by STING and induce type I IFN production.

## Adjuvanticity through DNA DAMPs

Although DNA DAMPs are closely associated with the development of autoimmune disease, DNA DAMPs also contribute to the activation of acquired immune responses following vaccination with alum adjuvant. Previous studies have shown that genomic DNA from dying cells induces the maturation of antigen-presenting cells as well as antigen-specific antibody and cytotoxic T cell responses. This suggests that self-DNA DAMPs can activate innate immune responses that induce acquired immunoresponses. Recently, Marichal et al. demonstrated that the adjuvanticity of alum was dependent on self-DNA released from cells at the alum inoculation site (Marichal et al., 2011). NLRP3 appears to be a key sensor in the induction of alum-mediated innate immunity, although its function is only partially dependent upon alum adjuvanticity. Intraperitoneal inoculation of mice with alum induced the recruitment of neutrophils, and the resulting alum deposits contained high amounts of genomic DNA. Because treatment with DNase I attenuated alum adjuvanticity, the alum-mediated release of genomic DNA may account for its potent adjuvanticity. In addition, the alum-mediated induction of antibody production is dependent on TBK1 and IRF3 as demonstrated using knockout mice, suggesting that alum-mediated genomic DNA induces high adjuvanticity of alum via the TBK1/IRF3 pathway, while alum-mediated uric acid production is less related to alum adjuvanticity via NLRP3 (Marichal et al., 2011). Furthermore, self-DNAs from alum inoculation can activate inflammatory monocytes, and homodimers of IL-12p40 are more important than type I IFN production upon alum adjuvanticity. Taken together, these findings suggest that self-DNA DAMPs are important for pathogen elimination, the development of autoimmune disease and the adjuvanticity of alum. Further analyses are required to elucidate which types of cells produce self-genomic DNA after adjuvant inoculation, and which sensors recognize extracellular genomic DNAs.

In addition to alum adjuvant, there are many licensed adjuvants such as MF59®, AS03®, and AS04®. Both MF59® and AS03® are emulsions of oil/water containing squalene. Although both adjuvants elicit antibody responses as well as cell-mediated immune responses specific for antigens, their mode of action has not been identified. Information on the receptors for and signaling induced by these adjuvants is needed, because unfortunate side effects can be expected more easily.

## Concluding remarks

Many sorts of nucleic acid species exist in the environment. These species affect all organisms such as the evolution of organisms, the inflammatory response, and the advent of drug-resistant microorganisms. To prevent pathogen infection, mammalian cells have equipped themselves with many sorts of sensors to recognize exogenous nucleic acid species as PAMPs, while those sensors are also stimulated by endogenous nucleic acids species as DAMPs. Dysfunction of the machineries sensing both PAMPs and DAMPs is strongly associated with chronic inflammatory disease or autoimmunity. In addition, both PAMPs and DAMPs underlie the action of vaccines, because most modern vaccines contain adjuvants, which are composed of both PAMP- and DAMP-associated molecules. Therefore, the machinery responsible for sensing nucleic acids species should be further elucidated to help us understand machinery of chronic infection, autoimmune development, identifying the side effects of vaccines, and developing safe vaccine adjuvants.

### Conflict of interest statement

The authors declare that the research was conducted in the absence of any commercial or financial relationships that could be construed as a potential conflict of interest.
